# Precious Stones; Early Notions Regarding Them

**Published:** 1891-07

**Authors:** 


### PRECIOUS STONES: EARLY NOTIONS REGARDING THEM.

In a queer old book about the value of precious stones, it is seriously stated that the swallowing of certain ones would prevent certain diseases.

The topaz was prescribed to stop bleeding wounds, and to make the heart light.

The diamond is advised for those who walk in their sleep and are slightly insane.

The loadstone would cure headaches, bites of serpents and deafness. Indeed, it is said to possess so many virtues that the head of any large family to-day is advised to buy one for each child, and in this way avoid the necessity of frequent calls and correspondingly long bills from a physician.

The sapphire makes the dull cheerful and brings perfection of health.

The opal is good for the eyes, for it causes the tears to flow.

Crystals prevent bad dreams, coral and cornelian stop hemorrhages.

Cardau, a great physician of olden times, writes most positively that the topaz, if hung around the neck, or swallowed in a drink, "will increase wisdom and repel fear." He also claimed to cure people of madness by making them swallow the yellow stone, and asserted that it never failed.

To the carbuncle, and to it alone, belongs the power of driving away devils. Under its guidance the evil spirit quietly departs, and nobody ever sees it go ; consequently nobody can deny its departure.

